# Biological characterization of D‐lactate dehydrogenase responsible for high‐yield production of D‐phenyllactic acid in *Sporolactobacillus inulinus*

**DOI:** 10.1111/1751-7915.14125

**Published:** 2022-08-03

**Authors:** Ya‐Yun Cheng, Tae Hyeon Park, Hyunbin Seong, Tae‐Jip Kim, Nam Soo Han

**Affiliations:** ^1^ Brain Korea 21 Center for Bio‐Health Industry, Development, Division of Animal, Horticultural, and Food Sciences Chungbuk National University Cheongju Korea

## Abstract

PLA (3‐D‐phenyllactic acid) is an ideal antimicrobial and immune regulatory compound present in honey and fermented foods. *Sporolactobacillus inulinus* is regarded as a potent D‐PLA producer that reduces phenylpyruvate (PPA) with D‐lactate dehydrogenases. In this study, PLA was produced by whole‐cell bioconversion of *S. inulinus* ATCC 15538. Three genes encoding D‐lactate dehydrogenase (*d‐ldh*1*, d‐ldh*2*,* and *d‐ldh*3) were cloned and expressed in *Escherichia coli* BL21 (DE3), and their biochemical and structural properties were characterized. Consequently, a high concentration of pure D‐PLA (47 mM) was produced with a high conversion yield of 88%. Among the three enzymes, D‐LDH1 was responsible for the efficient conversion of PPA to PLA with kinetic parameters of *Km* (0.36 mM), *k*_*cat*_ (481.10 s^−1^), and *k*_*cat*_ */Km* (1336.39 mM^−1^ s^−1^). In silico structural analysis and site‐directed mutagenesis revealed that the Ile307 in D‐LDH1 is a key residue for excellent PPA reduction with low steric hindrance at the substrate entrance. This study highlights that *S. inulinus* ATCC 15538 is an excellent PLA producer, equipped with a highly specific and efficient D‐LDH1 enzyme.

## Graphical abstract

*Sporolactobacillus inulinus* ATCC 15538 is a potential D‐phenyllactic acid producer. D‐LDH1 is the major enzyme for production of D‐phenyllactic acid from phenylpyruvate. Residue Ile307 in D‐LDH1 plays an important role in phenylpyruvate‐catalyzing.

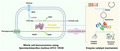

## INTRODUCTION

Phenyllactic acid (PLA), also known as 2‐hydroxy‐3‐phenyl‐propanoic acid, is a natural organic acid compound found in honey and fermented foods (Li et al., 2015). PLA plays an important role in the food industry as a natural preservative because it has no side effects on the human health (Liu et al., 2020). PLA exhibits an excellent antimicrobial activity against spoilage‐inducing and pathogenic microorganisms, such as yeasts, bacteria, and moulds, by damaging cell membrane and preventing biofilm formation (Chatterjee et al., 2017; Liu et al., 2021). Currently, PLA is considered a new generation drug that can be used to treat a wide range of diseases, such as type 2 diabetes and colitis (Ilavenil et al., 2015; Zhou et al., 2021). In addition, PLA acts as a potent regulator of immune function and energy homeostasis in humans by activating the G protein–coupled receptors for hydroxycarboxylic acids (Peters et al., 2019). Among the stereoisomers, D‐PLA exhibits better performance than l‐PLA in both antimicrobial activity and immune regulation (Mu et al., 2012c).

PLA can be produced by various lactic acid bacteria (LAB), such as *Lactobacillus*, *Weissella*, *Pediococcus*, and *Leuconostoc* (Mu et al., 2012a; Ndagano et al., 2011; Zheng et al., 2011). LAB are generally recognized as safe (GRAS) microorganisms that have many applications in the food industry to guarantee food safety, improve food flavour, and enhance health‐promoting activity (Levit et al., 2021). In LAB, PLA is synthesized via the amino acid metabolic pathway, in which phenylalanine is catabolized to phenylpyruvate (PPA) by transaminase and then reduced to PLA by lactate dehydrogenase (LDH; Yang et al., 2019). LDH is the enzyme that plays a crucial role in the final production of PLA.

In general, LAB possess multiple LDH isozyme genes, and their substrate specificities vary depending on the bacterial species and isozymes (Chen et al., 2017). LDH enzymes produce stereoisomeric forms of products, such as D‐forms or L‐forms, and their genetic or protein sequences vary depending on their specificity. For instance, the *lrldh* gene of *Lactobacillus rossiae* is responsible for D‐PLA production, whereas the *l‐ldh* gene of *Lactiplantibacillus plantarum* is responsible for L‐PLA production (Luo et al., 2020; Zhu et al., 2017). Considering the higher antimicrobial and immune‐regulatory activities of D‐PLA, bacteria with highly active *d*‐*ldh* genes are suitable biocatalysts for large‐scale production of D‐PLA (Sakurai et al., 2021).

Recently, *S. inulinus* has attracted the attention of researchers due to its superior productivity of D‐lactate (>180 g L^−1^) with high optical purity (Reddy Tadi et al., 2017; Zhao et al., 2014). Wu et al. (2019) reported that three D‐LDHs and two L‐LDHs exist in the chromosome of *S. inulinus* CASD and that these enzymes have different activities for pyruvate reduction. Meanwhile, a D‐lactate dehydrogenase named DLDH744 from *S. inulinus* CASD was found to have sufficient enzymatic activity to produce PLA with low efficiency (*k*_*cat*_ */Km* as 1.49 ± 0.076 mM^−1^ s^−1^; Wang et al., 2016). However, the PLA production capacity of *S. inulinus* and the enzymes responsible for this reaction have not yet been investigated in detail. Considering the shared enzymatic activity of lactate dehydrogenase against pyruvate or PPA, we hypothesized that *S. inulinus* would have specific D‐LDHs that can produce either D‐PLA or D‐lactate.

In this study, we aimed to investigate the enzymatic and structural characteristics of the D‐LDHs responsible for PLA production in *S. inulinus*. We initially demonstrated high‐level conversion of D‐PLA from PPA using whole‐cells of *S. inulinus* ATCC 15538. We then cloned and expressed three genes encoding D‐LDHs in *Escherichia coli* and performed biochemical and in silico structural analyses of these enzymes. Finally, we conducted the site‐directed mutagenesis to determine the key amino acid residue of the D‐LDH1 enzyme for PLA production. Our current finding will provide the basic information to develop not only industrial microorganisms but also efficient enzymes for D‐PLA overproduction.

## EXPERIMENTAL PROCEDURES

### Strains, plasmids, and primers

The bacterial strains, plasmids, and primers used in the present study are listed in Table 1. *S. inulinus* ATCC 15538 was grown at 37°C in GYP medium, which was composed of 2% glucose, 1% yeast extract, 1% peptone, 1% sodium acetate, and 0.5% (v/v) salt solution (4% MgSO_4_·7H_2_O; 0.16% MnSO_4_·4H_2_O; 0.2% FeSO_4_·7H_2_O; and 0.2% NaCl; pH 6.8; Chang et al., 2008). *E. coli* DH5α and *E. coli* BL21 (DE3) strains were used for cloning and protein expression, respectively, and they were grown aerobically at 37°C in Luria–Bertani (LB) medium. The *E. coli* transformants were grown in LB medium containing 100 μg ml^−1^ ampicillin at 37°C. The pET‐21a (+) plasmid (Novagen) was used for DNA manipulation.

**TABLE 1:** Comparative analysis of phenyllactate production from wild‐type microorganism reported in the literatures

Microorganism	Substrate	Method	Production process	Chirality	Production (mM)	Productivity (mM h^−1^)	Yield (%)	References
*Sporolactobacillus inulinus* ATCC *15538*	PPA	Whole‐cell bioconversion	Batch	D‐PLA	47.00	94.00	88.00	This study
*Bacillus coagulans* SDM	PPA	Whole‐cell bioconversion	Fed‐batch	Racemic PLA	224.47	13.84	70.00	Zheng et al. (2011)
*Lactobacillus* sp. SK007	PPA	Fermentation	Fed‐batch	Racemic PLA	104.59	1.45	51.10	Mu et al. (2009)
*Lactobacillus* sp. SK007	PPA	Whole‐cell bioconversion	Batch	Racemic PLA	6.80	1.70	56.67	Li et al. (2007)
*Pediococcus pentosaceus* SK25	MRS	Fermentation	Batch	Racemic PLA	0.82	0.02	6.80	Yu et al. (2015)
*Lactobacillus crustorum* NWAFU *1078*	PPA	Whole‐cell bioconversion	Batch	Racemic PLA	15.20	7.60	76.00	Xu et al. (2020)
*Leuconostoc mesenteroides* ATCC 8293	PPA	Whole‐cell bioconversion	Batch	D‐PLA	24.66	8.22	83.30	Li et al. (2014)
	PPA	Fermentation	Batch	D‐PLA	27.50	1.53	68.75	

### Bioconversion for PLA production

*Sporolactobacillus inulinus* ATCC 15538 was grown in GYP medium at 37°C under microaerobic conditions at 150 rpm, and the late exponential phase cells were collected after 16 h by centrifugation at 10,000 *g* for 20 min. The cell pellet was washed twice with 0.85% NaCl and resuspended in 50 mM Tris–HCl buffer for bioconversion. The bioconversion conditions were set as follows: 0.02 g ml^−1^ of the cell pellet (dry cell mass) was resuspended in 50 mM Tris–HCl buffer (pH 8.0) with 70 mM PPA and 275 mM glucose; bioconversion was performed at 30°C at 150 rpm for 3 h.

### Cloning, expression, and purification of D‐LDHs from *S. inulinus*

Using the primer sets described in Table S1, three D‐LDH genes were amplified from the genomic DNA of *S. inulinus* ATCC 15538 using PCR, and the PCR fragments were purified using the AccuPrep® PCR purification kit (Bioneer Inc.). The purified PCR products were ligated with pET‐21a (+) by the homologous recombination of primer sets that shared complementary sequences, using the In‐Fusion® HD cloning kit (Takara). All recombinant plasmids were transformed into *E. coli* DH5α competent cells, and the transformants were selected on LB medium containing 100 μg ml^−1^ ampicillin. Sequences of the recombinant plasmids were analysed using a high‐throughput DNA analyser (Cosmo Genetech Inc.). The recombinant plasmids were transformed into *E. coli* BL21 (DE3) cells for protein expression. The *E. coli* BL21 (DE3) transformants were cultured in LB broth with 100 μg ml^−1^ ampicillin at 37°C until optical density reached 0.6 at 600 nm (Chatterjee et al.). Subsequently, protein expression was induced at 20°C with shaking at 150 rpm for 12 h and by addition of 0.1 mM isopropyl‐β‐D‐thiogalactopyranoside (IPTG; Zheng et al., 2017). Cells were harvested by centrifugation at 7000 *g* for 10 min and resuspended in lysis buffer (10 mM imidazole, 500 mM sodium chloride, and 20 mM sodium phosphate; pH 7). The cells were disrupted by sonication (VP‐050; TAITEC, Nishikata Koshigaya), and soluble enzymes were obtained by centrifugation at 7000 *g* for 10 min. Gravity‐flow affinity chromatography was used for His‐tagged enzyme purification using an Ni‐NTA agarose column (QIAGEN). The bound enzymes were eluted using elution buffer (300 mM imidazole, 500 mM sodium chloride, and 20 mM sodium phosphate; pH 7). Desalting and concentration of enzyme analysis were performed using Vivaspin® 500 centrifugal concentrators (10,000 MWCO, Sartorius). Protein molecular masses were measured using sodium dodecyl sulfate‐polyacrylamide gel electrophoresis (SDS‐PAGE).

### Enzymatic activity assay

After purification, the reduction activity of D‐lactate dehydrogenase on PPA and pyruvate was assayed photometrically (absorbance at 340 nm) by measuring the decrease in NADH. Briefly, the enzyme reaction was performed in 96‐well plates with 300 μl solution (0.3 mM NADH, 10 mM sodium pyruvate, or sodium phenylpyruvate in 100 mM sodium phosphate buffer, pH 7.0) and 10 μl of properly diluted enzyme solution (Cook et al., 2015). One unit of enzyme activity was defined as the amount of enzyme required to oxidize 1 μM NADH per minute. Protein concentration was measured using the Pierce™ BCA protein assay kit (Thermo Fisher Scientific Inc.), with bovine serum albumin (BSA) as the standard.

### Site‐directed mutagenesis of D‐LDH1

Site‐directed mutagenesis was performed using pET‐21a‐D‐LDH1 as a template. The nucleotide sequences of mutagenic primers are listed in Table S1. To obtain a linearized template DNA for the mutagenesis of D‐LDH1, PCR amplification was performed with a pair of primers (pELDH1‐F and pELDH1‐R) using pESiLDH1 as a template. Using the resulting PCR product as a template, a second PCR was performed with two different sets of mutagenic primers (D‐LDH1_I307M‐F/D‐LDH1_I307M‐R and D‐LDH1_I307L‐F/D‐LDH1_I307L‐R). PCR amplification was performed with Pyrobest™ DNA polymerase (TaKaRa Bio Inc.) using a thermal cycler C1000™ (Bio‐Rad Inc.) as follows: 30 cycles of denaturation (10 s at 98°C), annealing (30 s at 61°C), extension (8 min 30 s at 72°C), and a final cycle of 8 min 30 s at 72°C. The resulting PCR products were purified using the *Accu*Prep PCR/Gel Purification Kit (Bioneer Co.), which was self‐ligated using the Overlap Cloner Kit (Elpis Biotech Co.) to generate the mutant plasmids pESiLDH1_I307M and pESiLDH1_I307L.

### In silico structural analysis of D‐LDHs

In the whole‐genome sequence of *S. inulinus* NBRC 13595 from the NCBI database, three D‐LDH genes (GenBank accession no. AFVQ02000236.1, AFVQ02000016.1, and AFVQ02000028.1) were found on the chromosome. To carry out primary structure analysis of these enzymes, amino acid sequences were retrieved from the NCBI database, and the amino acid sequences which have high similarity with D‐LDH from *S. inulinus* were collected using the BLASTP online service. Subsequently, multiple sequence alignment was performed using ClustalX2 software, and phylogenetic analysis was performed by the neighbour‐joining method using MEGA v7.0 (Han et al., 2019). In addition, the secondary structures of these enzymes were analysed based on the aligned sequences using ESPript3. The amino sequences of LDH with high pyruvate reductase activity (LEUM_1756) from *Leuconostoc mesenteroides* ATCC 8293 and higher PPA reductase LDH (MH920335) from *Pediococcus claussenii* were used for comparison. Homology models of D‐LDH1 and D‐LDH2 were constructed using the SWISS‐MODEL online server based on the X‐ray crystal structures of D‐LDH from *S. inulinus* CASD (PDB ID: 4XKJ) and *Pseudomonas aeruginosa* PAO1 (PDB ID: 3WWZ), respectively (Nadeem et al., 2018). Crystal structure alignment and protein 3D structure visualization and editing were performed using PyMOL software (Meng et al., 2016). The predicted structural proteins were docked with pyruvate and PPA using Schrodinger software (Jung et al., 2016).

### Chemical analysis methods

A high‐performance liquid chromatography (HPLC) system (Agilent 1206 Infinity, Agilent Technologies) equipped with a UV absorbance detector was used to detect the D−/L‐PLA isomers produced by D‐LDHs. D−/L‐PLA isomers were measured using a chiral ORpak CRX853 column (8.0 × 50 mm, Shodex, Showa Denko) coupled with a CRX‐G column on the same HPLC system at 250 nm with 1 mM CuSO_4_ as the solvent at a flow rate of 1 ml min^−1^ at 25°C (Jin et al., 2016).

### Statistical analysis

All experiments were performed in triplicate. Sigmaplot was used for data analysis, and statistical significance analysis was performed using IBM SPSS Statistics software (version 25).

## RESULTS

### Production of PLA by whole‐cell bioconversion

**FIGURE 1:**
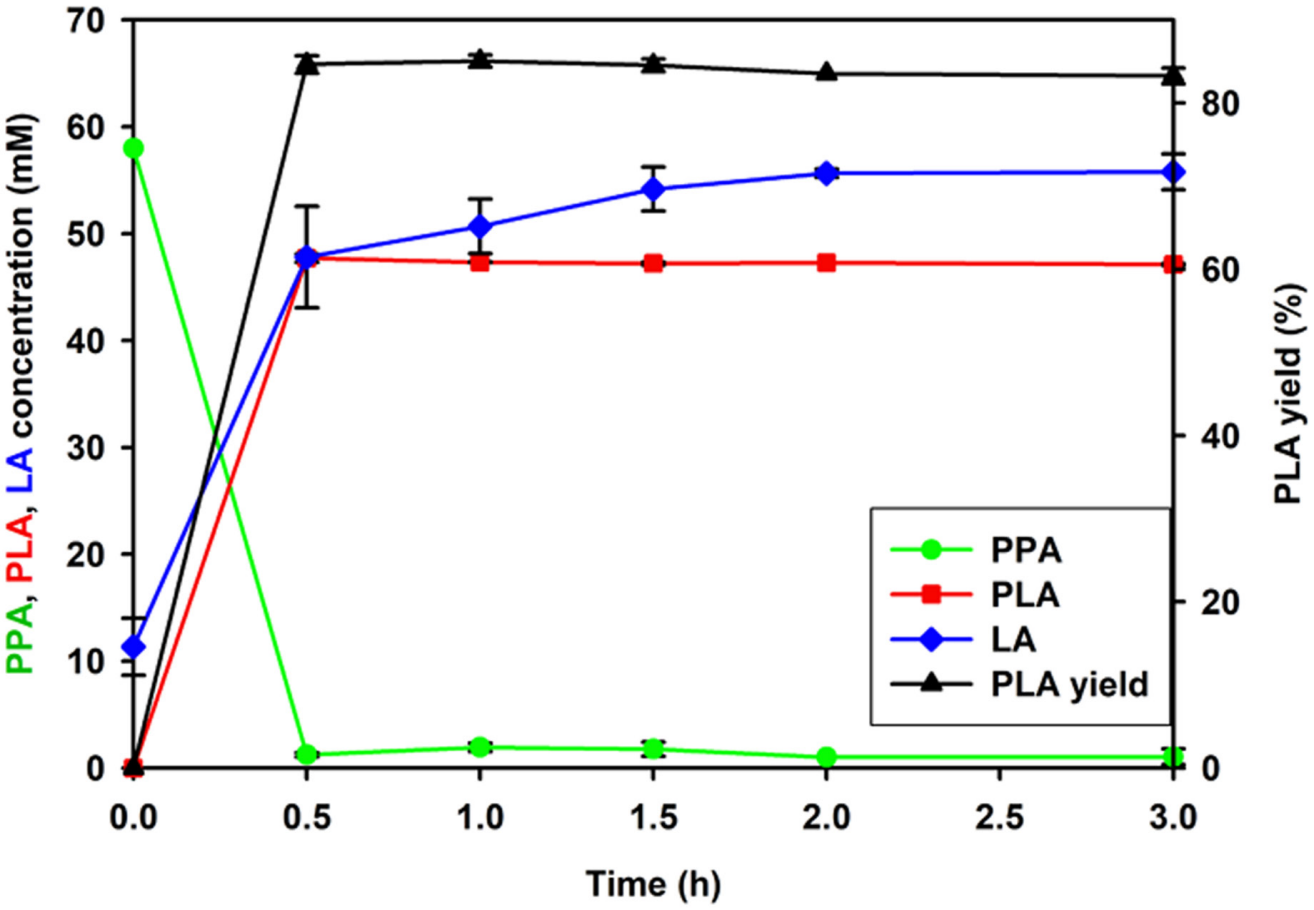
Time course of the batch bioconversion of phenyllactic acid (PLA) from phenylpyruvate (PPA) using whole‐cells of *Sporolactobacillus inulinus* ATCC 15538. The concentrations of PLA, PPA, and lactic acid (LA) were measured during the biocatalytic reaction using whole‐cells of *S. inulinus* (0.02 g mL^−1^ freeze‐dried cell mass) in 50 mM tris–HCl buffer (pH 8) containing 70 mM PPA and 275 mM glucose at 30°C for 3 h.

Bioconversion of PPA to PLA by *S. inulinus* was performed under optimal conditions for 3 h. As shown in Figure 1, PPA was rapidly consumed with the accumulation of PLA in 0.5 h, and then the PLA concentration and conversion yield reached maximum levels of 47 mM and 88%, respectively. When the stereo forms of PLA product were analysed, pure D‐PLA was produced during the bioconversion process (Figure S1). In addition, the PLA production from wild type microorganisms is listed in Table 1. Compared with other microorganisms, *S. inulinus* ATCC 15538 shows highest productivity (94 mM·h^−1^) and PPA conversion yield (88%). Also, pure D‐PLA was produced by *S. inulinus* ATCC 15538 instead of racemic PLA produced by *Bacillus coagulans*, *Lactobacillus*. sp. SK007, *Pediococcus pentosaceus* SK25, and *Lactobacillus crustorum* NWAFU 1078. These results show that *S. inulinus* ATCC 15538 has superior properties for D‐PLA production, with a rapid conversion rate (94 mM·h^−1^) and high conversion yield (88%).

### Phylogenetic analysis

**FIGURE 2:**
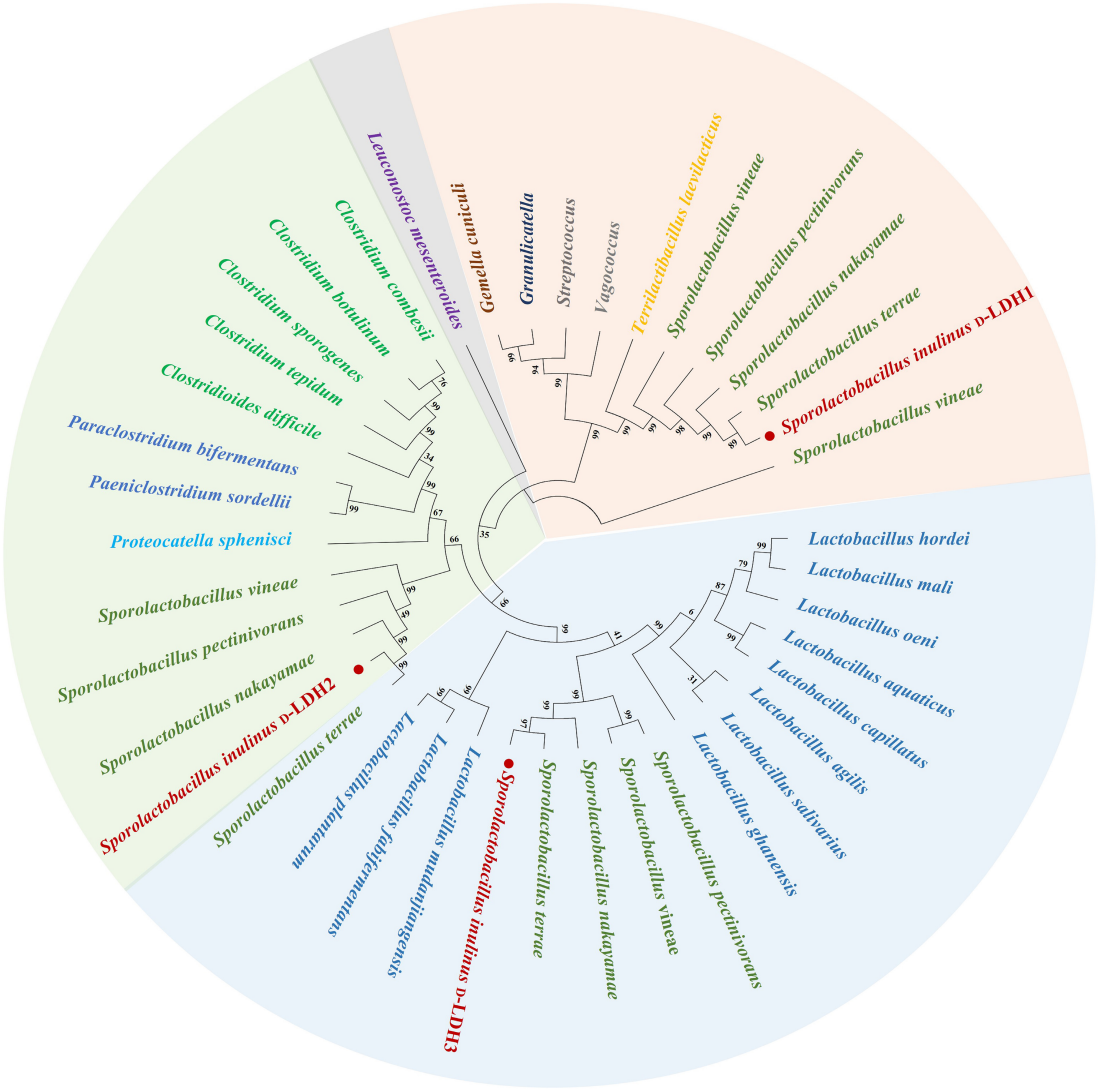
Phylogenetic tree of D‐lactate dehydrogenases (LDH) from various microorganisms based on their amino acid sequences. The three D‐LDHs from *Sporolactobacillus inulinus* ATCC 15538 denoted in red belong to the three different clusters in orange, green, and blue, respectively, showing their low level of homology.

To investigate the evolutionary relationship between D‐LDHs in the chromosome of *S. inulinus* ATCC 15538 and those from other microorganisms, a phylogenetic tree of D‐LDHs was constructed based on their amino acid sequences using the neighbour‐joining method (Figure 2). As a result, three D‐LDHs from *S. inulinus* ATCC 15538 belonging to the D‐2‐hydroxy acid dehydrogenase family were clustered into three different clades, showing their difference in amino acid sequences, indicating that they play different roles in the process of cell growth. D‐LDH1 was categorized along with *Paeniclostridium*, *Clostridioides*, *Paraclostridium*, *Clostridium*, and *Proteocatella*. D‐LDH2 was grouped within the genera *Vagococcus*, *Terrilactibacillus*, *Streptococcus*, *Granulicatella*, and *Gemella*. Notably, D‐LDH3 was clustered into a monophyletic group with *Lactobacillus*. These three D‐LDHs of *S. inulinus* were commonly present in other species of *Sporolactobacillus*, and their similarity levels were in the order of *S. terrae* > *S. nakayamae* > *S. pectinivorans*. This result indicated the conserved presence of three D‐LDH genes in the genus of *Sporolactobacillus*.

### Cloning, expression, and purification of D‐LDHs

**FIGURE 3:**
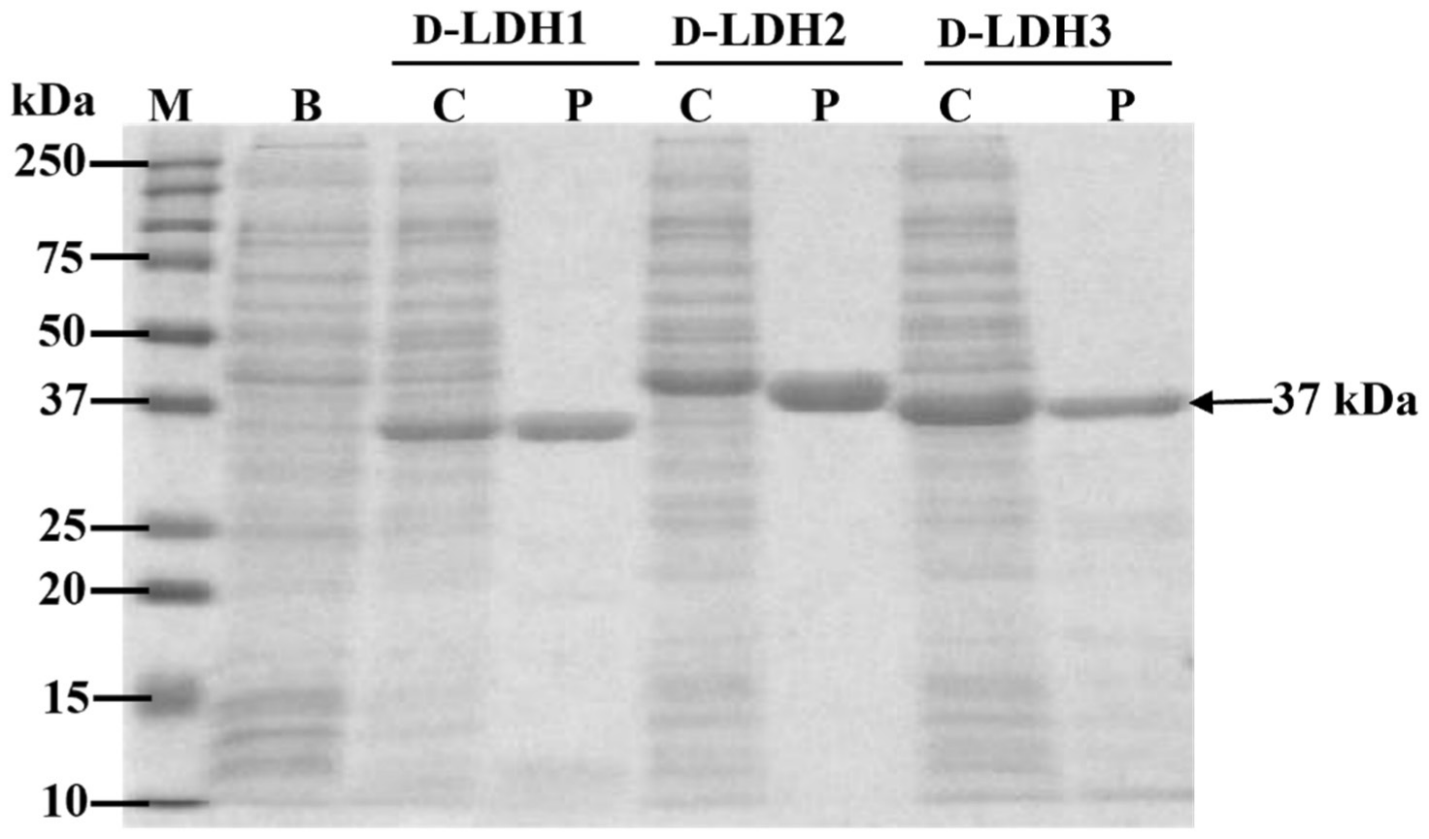
SDS‐PAGE results of the *Escherichia coli* BL21 star (DE3) recombinants. M, protein marker; B, blank, crude cell extract of *E. coli* BL21 (DE3) transferred with pET‐21a (+); C, crude cell extract of D‐LDHs; P, purified cell extract of D‐LDHs using Ni‐NTA column chromatography.

Three D‐LDH genes (*d*‐*ldh*1, *d‐ldh*2, and *d*‐*ldh*3) from *S. inulinus* ATCC 15538 were cloned into a pET‐21a (+) vector because pure D‐PLA was produced shown in Figure 1. The open‐reading frames of the *ldh* genes were 1026, 1005, and 1008 bp, encoding 341, 334, and 335 amino acids, respectively. The expression of *d*‐*ldh*1, *d‐ldh*2, and *d*‐*ldh*3 in recombinant transformants was analysed by SDS‐PAGE (Figure 3). Thick bands were observed in the crude extract of recombinant *E. coli*. After purification by Ni‐NTA affinity chromatography, single bands were clearly observed revealing purification to homogeneity

### Kinetic analysis of D‐LDHs

Kinetic analyses of the three D‐LDHs was performed using various concentrations of PPA and pyruvate. As shown in Table 2, the kinetic parameters were determined using a Lineweaver–Burk plot, and the results were compared with those data reported in the previous studies (Lee et al., 2019; Mu et al., 2012a; Tokuda et al., 2003; Yu et al., 2012). D‐LDH1 showed the highest *k*_*cat*_ */Km* value (1336.39) for PPA and a lower enzymatic efficiency for pyruvate (*k*_*cat*_ */Km*, 0.25). Meanwhile, D‐LDH2 showed the highest value of *k*_*cat*_ */Km* (219.87) for pyruvate, and the lowest value of *k*_*cat*_ */Km* value for PPA was 0.05. Moreover, D‐LDH3 showed low activity against both PPA (*k*_*cat*_ */Km* = 0.20) and pyruvate (*k*_*cat*_ */Km* = 0.96). This result revealed the efficient reducing activity of D‐LDH1 for PPA (*Km* = 0.36 mM and *K*_*cat*_ = 481.10 s^−1^). When the enzyme efficiencies were compared based on published data, both pc‐HADH from *Pediococcus claussenii* and D‐LDH1 from *S. inulinus* showed significantly high values (1348 and 1336.39 mM^−1^ s^−1^, respectively) of *k*_*cat*_ */Km* than others. Moreover, D‐LDH1 from *S. inulinus* exhibited the highest value of *k*_*cat*_ */Km*_(PPA)_ */k*_*cat*_ */Km*_(pyruvate)_ at 534556, indicating the highest substrate specificity for PPA among the homologous enzymes tested

**TABLE 2:** Kinetic analysis results of three D‐lactate dehydrogenases of *Sporolactobacillus inulinus* ATCC 15538

Microorganisms	Enzymes	Substrates						$\frac{k\mathrm{cat}/Km\;\left(\mathrm{PPA}\right)}{k\mathrm{cat}/Km\;\left(\text{Pyruvate}\right)}\times 100\left(\%\right)$	References
		Pyruvate			Phenylpyruvate				
		*Km* (mM)	*k*_cat_ (S^−1^)	*k*_cat_/*km* (mM^−1^ S^−1^)	*Km* (mM)	*k*_cat_ (S^−1^)	*k*_cat_/*km* (mM^−1^ S^−1^)		
*Sporolactobacillus inulinus* ATCC 15538	D‐LDH1	21.45	5.28	0.25	0.36	481.10	1336.39	534,556.00	This study
	D‐LDH2	1.52	313.76	219.87	0.38	0.02	0.05	0.02	This study
	D‐LDH3	1.57	0.30	0.20	0.40	0.41	0.96	480.00	This study
Mutants	D‐LDH1 I307L	24.29	10.93	0.45	0.92	676.13	733.27	162,948.00	This study
	D‐LDH1 I307M	3.48	14.91	4.29	1.54	68.96	44.91	1046.85	This study
*Sporolactobacillus inulinus* CASD	DLDH744	3.4	8.85	2.6	3.32	4.94	1.49	57.31	Wang et al. (2016)
	DLDH744 M307L	4.75	6.05	1. 20	1.27	8.29	6.53	544.17	
*Bacillus coagulans* SDM	D‐nLDH	2.20	23.6	11	4.40	16.5	3.9	35.45	Zheng et al. (2011)
*Lactobacillus pentosus* JCM1558	D‐LDH	0.12	321	2675	0.8	40	50	1.87	Tokuda et al. (2003)
*Leucconostoc mesenteroides* ATCC 8293	LEUM_1756	0.58	2900	5000	15.4	5646	366.62	7.53	Li et al. (2014)
*Pediococcus pentosaceus* ATCC 25745	D‐LDH	0.49	320	658	1.73	173	100	15.20	Yu et al. (2012)
*Pediococcus acidilactici* DSM 20284	D‐LDH	0.09	287	3157	2.90	305	105	3.33	Mu et al. (2012a)
*Oenococcus oeni*	oo‐HADH	1.12	674	602	15.6	77.8	5	0.83	Lee et al. (2019)
*Weissella confusa*	wc‐HADH	1.28	1434	1124	20.3	457	22.5	2.00	
*Pediococcus claussenii*	pc‐HADH	0.56	459	813	0.35	472	1348	165	

### In silico structural analysis of the D‐LDHs

**FIGURE 4:**
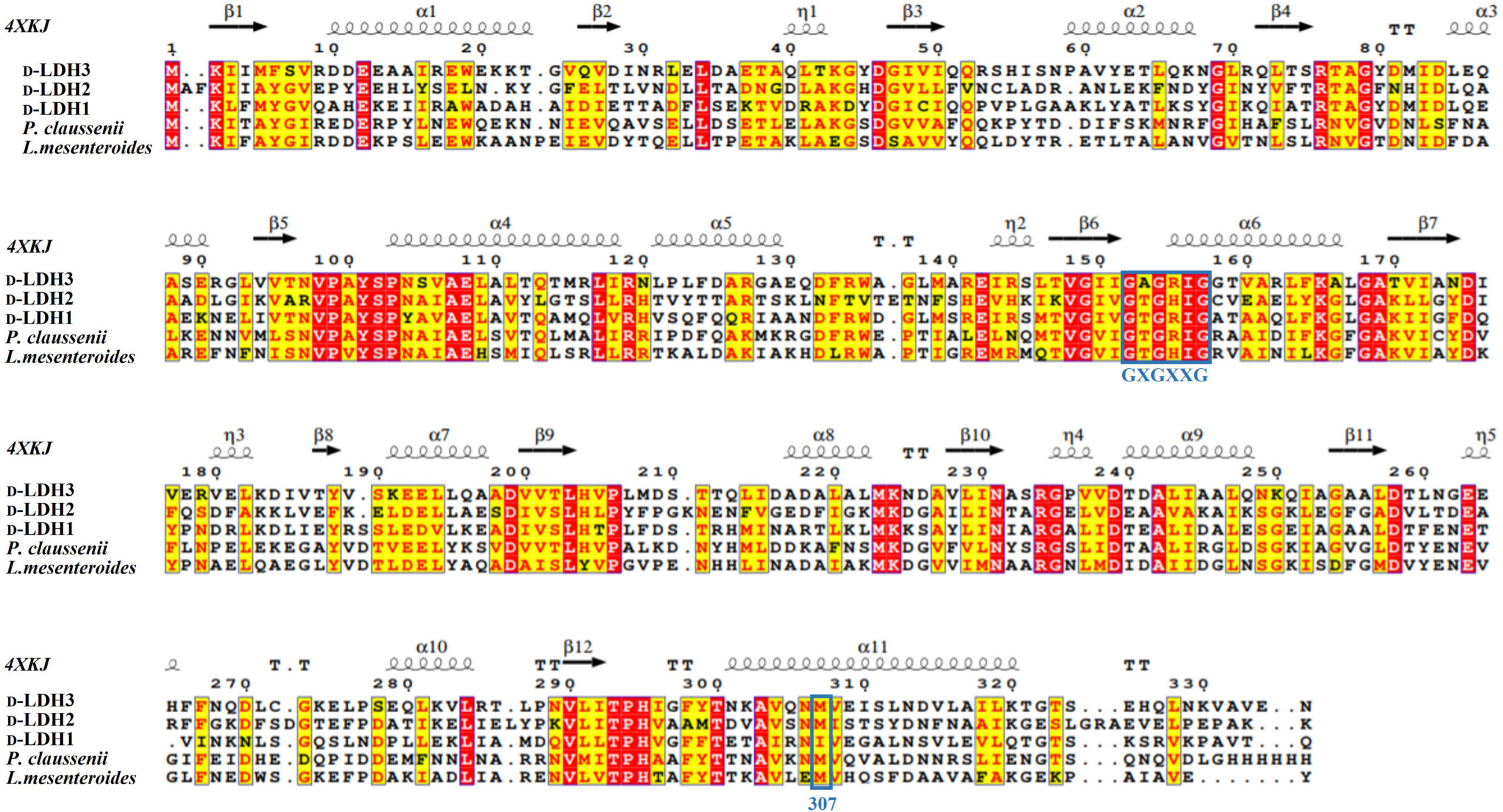
Amino acid sequence alignment of D‐LDHs from *Sporolactobacillus inulinus* ATCC 15538 with homologous enzymes. The secondary structure of D‐LDH from *S. inulinus* CASD (PDB ID 4XKJ) is presented at the top. Helices are marked with spirals, beta‐strands with arrows, and turns with the letter T. identical residues and conserved substitutions are shaded in red. The nucleotide‐binding signature domain GXGXXG is identified by the blue square. Ile307 is a unique residue in D‐LDH1 that differs from residues in the same position as the other D‐lactate dehydrogenases.

**FIGURE 5:**
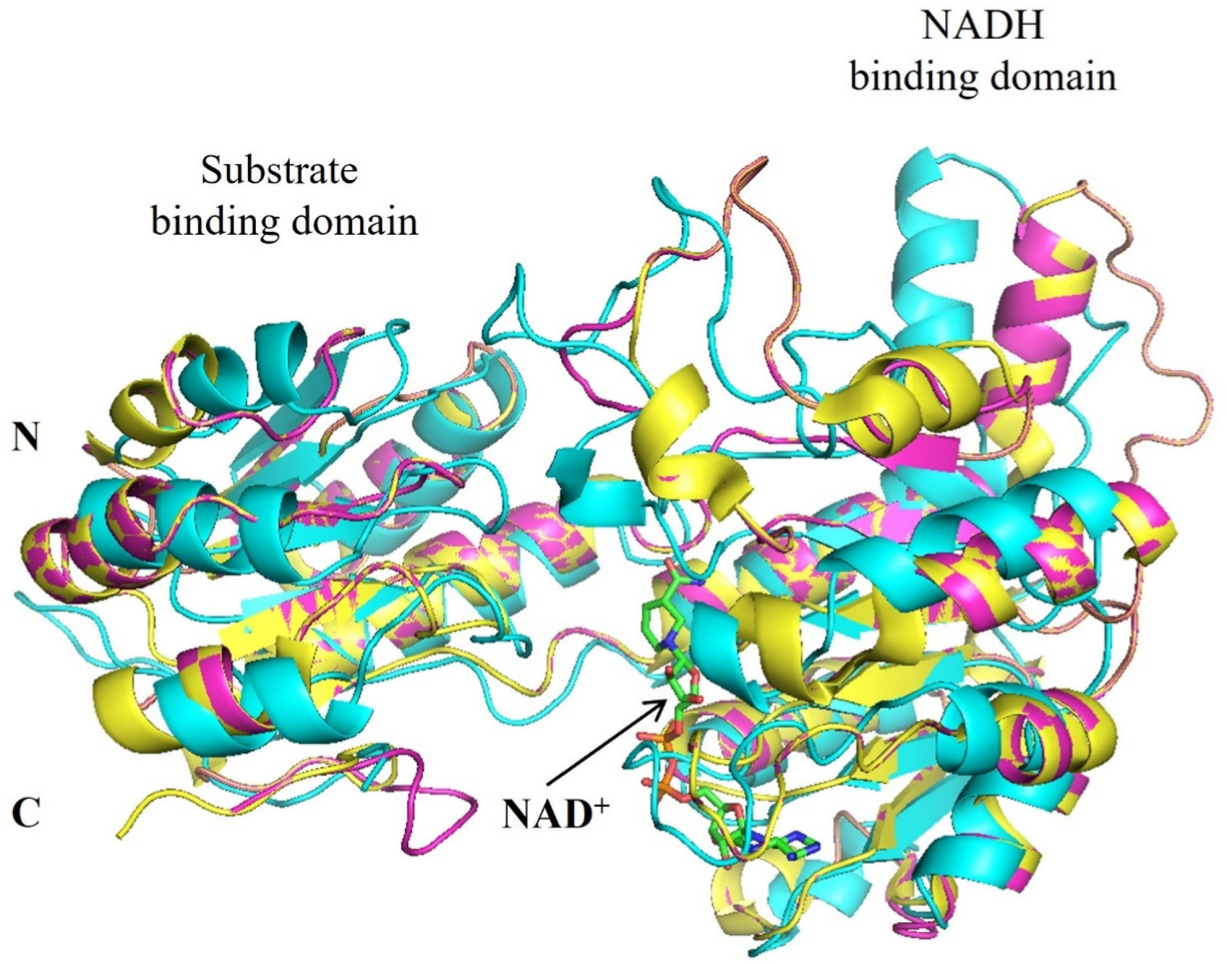
Tertiary structure alignment of three D‐lactate dehydrogenases, D‐LDH1 (pink), D‐LDH2 (cyan), and D‐LDH3 (yellow) of *Sporolactobacillus inulinus* ATCC 15538. The carbon atoms of NADH are shown in green.

To characterize these three enzymes, their amino acid sequences, secondary structures, and tertiary structures were aligned (Figures 4 and 5). As shown in Figure 4, the conserved sequence GXGXXG (where X represents any of the 20 amino acids) for a dinucleotide‐binding motif appears in all D‐LDHs at positions 151–157 (Vinals et al., 1993). D‐LDH1 showed higher sequence similarity with D‐LDH3 (48.48%) compared to D‐LDH2 (36.74%). The tertiary structures of D‐LDHs (Figure 5) consist of both a substrate‐binding domain and a cofactor‐binding domain with a deep cleft between these two domains. The active site is located in the deep cleft, where its size and shape determine the difficulty of the substrate to enter the pocket, thereby affecting the substrate specificity and catalytic properties of D‐LDHs (Fan et al., 2021). D‐LDH1 and D‐LDH3 share similar structural patterns in both the substrate‐binding domain and cofactor‐binding domains, respectively. D‐LDH2 displays a dislocation structure, implying the different enzymatic characteristics of D‐LDH2

### Exploration of the key residue for PPA reduction

**FIGURE 6:**
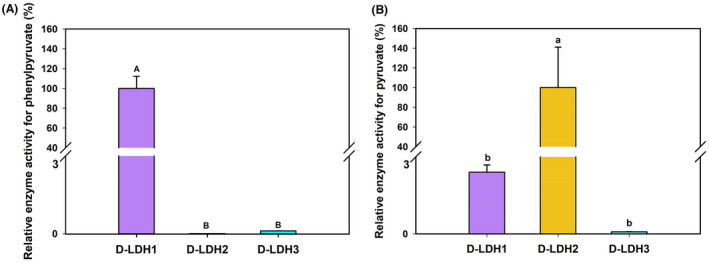
Relative enzymatic activities of D‐LDHs on phenylpyruvate (A) and pyruvate (B). Significant difference is indicated by letters. Lowercase letter, *p* < 0.05; uppercase letter, *p* < 0.01.

**FIGURE 7:**
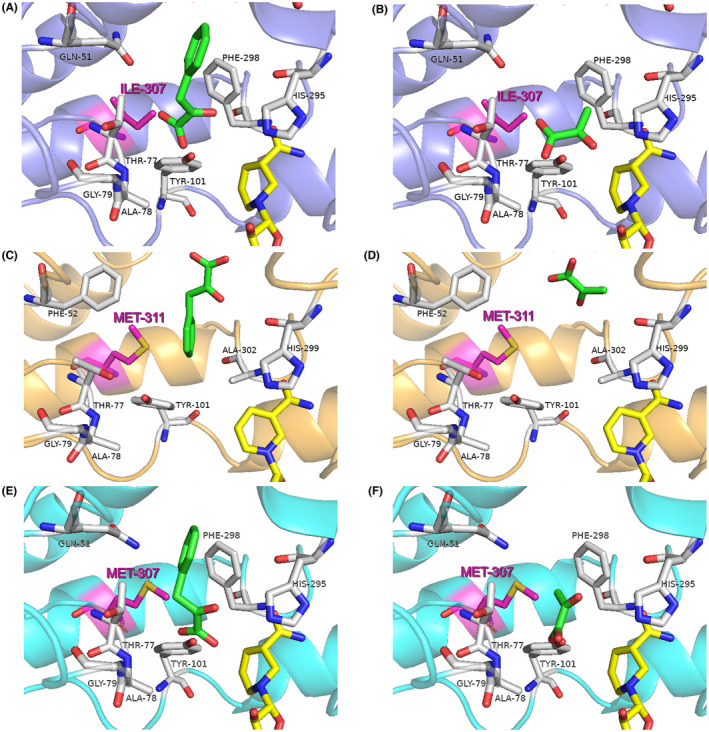
Active sites of the D‐LDHs from *Sporolactobacillus inulinus* ATCC 15538 docked with substrates. (A), D‐LDH1 with PPA; (B), D‐LDH1 with pyruvate; (C), D‐LDH2 with PPA; (D), D‐LDH2 with pyruvate; (E), D‐LDH3 with PPA; (F), D‐LDH3 with pyruvate. The carbon atoms of NADH are shown in yellow, substrate docking poses are shown in green, active site residues are shown in grey, and the key residue 307 is shown in pink.

To identify the key amino acid residues for binding with PPA in D‐LDHs, the relative enzyme activities toward PPA and pyruvate were investigated (Figure 6), and structure modelling and substrate docking were performed (Figure 7). As shown in Figure 6, D‐LDH1 exhibited the highest enzyme activity towards PPA, D‐LDH2 exhibited the highest enzyme activity towards pyruvate, and D‐LDH3 showed low enzyme activity towards both PPA and pyruvate, which was consistent with the kinetic data. In D‐LDH1, the residues Gln9, Gln51, Asn268, and Ile307 are located around PPA, and the residues Gly79 and Tyr101 are involved in binding with PPA by hydrogen bonds (Wang et al., 2016). In addition, the aromatic ring of the Phe298 residue appears to bind to the aromatic ring of PPA via a π–π interaction. In the case of D‐LDH2, the Phe residue was replaced with Ala at residue 298. Therefore, we hypothesized that the absence of π–π interaction with the aromatic ring of PPA caused the loss of most PPA reducing activity in Ala298 of D‐LDH2. In addition, D‐LDH2 has a larger space in the substrate‐binding pocket, which may result in higher substrate affinities for PPA or PA (low *Km* values in Table 2). Notably, D‐LDH3 showed a binding mode similar to that of D‐LDH1, except for residue Met307 (Ile307 in D‐LDH1), whereas its enzyme activity for PPA reduction was much lower than that of D‐LDH1. Accordingly, amino acid residue 307 is thought to play an important role in PPA reduction.

When the D‐LDH enzyme catalyses the reaction, its three‐dimensional structures for substrate‐binding sites determine its affinity for PPA or pyruvate. In previous studies on D‐LDHs, it was found that His295, Arg234, and Glu263 were catalytic triads and the side chains of Thr77 and Tyr101 and the nitrogen atoms of Ala78 were responsible for binding with the carboxylate group of the PPA molecule. In addition, Gly79 was located at the entrance of the substrate binding site, Tyr101 and Met307 stayed inside the substrate entry pathway, and they were highly conserved in D‐α‐hydroxy acid dehydrogenases (Fan et al., 2021; Wang et al., 2016). The amino acid residues related to substrate entrance and binding were highly conserved in D‐LDHs; however, Met307 was substituted by Ile 307 in D‐LDH1. Therefore, Ile307 appeared in D‐LDH1 instead of Met307 in D‐LDH3, which increased the probability of achieving the highest PPA reduction activity in D‐LDH1 due to its relatively short chain. In general, residues with large and long side chains can decrease the binding affinity of PPA molecules due to steric hindrance, thereby affecting substrate specificity and enzyme activity.

### Mutagenesis analysis

**FIGURE 8:**
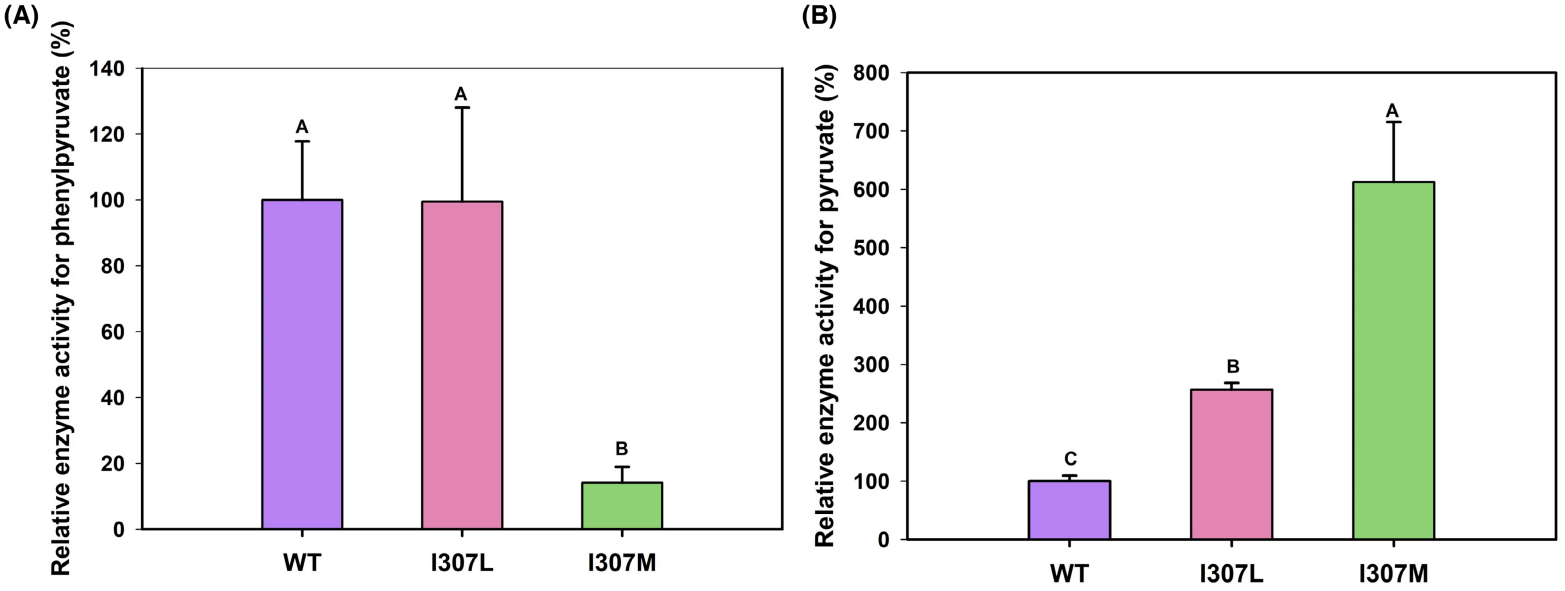
Relative enzymatic activities of wild‐type D‐LDH1 and its mutants on phenylpyruvate (A) and pyruvate (B). Significant difference (*p* < 0.01) is indicated by letters. WT, wild‐type of D‐LDH1; I307L, D‐LDH1 mutant I307L; I307M, D‐LDH1 mutant I307M.

To investigate the role of Ile307 residue in D‐LDH1 in PPA reduction, the amino acid residue Ile307 was substituted with Leu or Met by site‐directed mutagenesis. As shown in Figure 8A, wild‐type D‐LDH1 and I307L showed the highest enzyme activity on PPA, whereas mutant I307M lost over 80% activity. In contrast, when measured towards pyruvate (Figure 8B), the enzyme activities of I307L and I307M were increased significantly (two‐ and five‐folds, respectively) compared to wild type D‐LDH1.

A similar trend was observed for the kinetic data (Table 2). Compared with wild‐type D‐LDH1, I307M had higher *Km* and lower *k*_*cat*_ values for PPA, but lower *Km* and higher *k*_*cat*_ values for pyruvate. In detail, for PPA reduction, the *k*_*cat*_ */Km* of I307M was decreased by 26‐folds and the *Km* of I307M was increased by approximately four‐folds compared with that of the wild type. Also, the single‐site mutation of M307L in D‐LDH3 has been done by Min Wang at el.’ study (Wang et al., 2016). As result, M307L of D‐LDH3 shown the increasement enzyme activity towards bulkier substrate PPA than the wild type. In a word, those results indicated that the amino acid residue Ile307 in D‐LDH1 is important for both PPA binding and reduction reactions.

**FIGURE 9:**
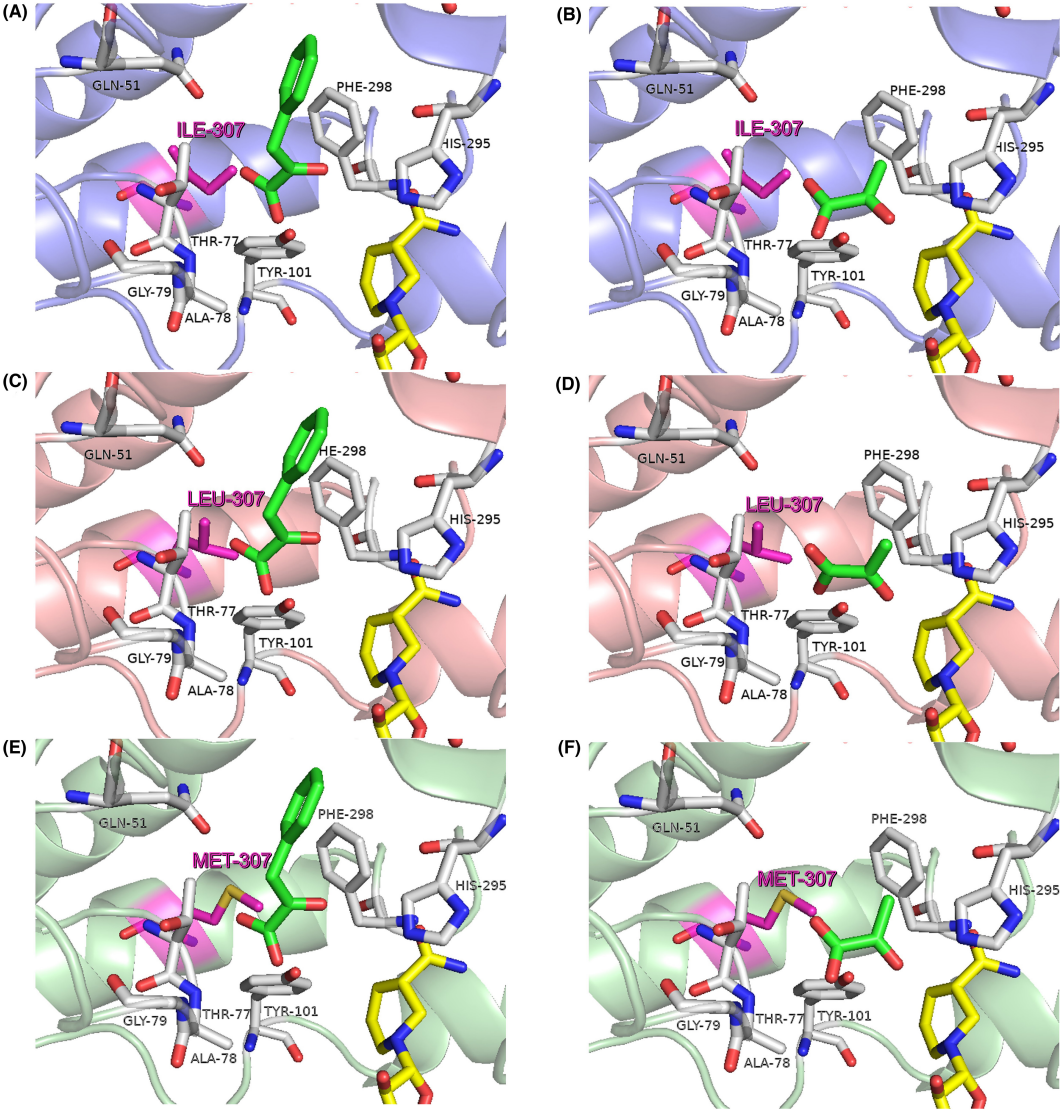
Active site of D‐LDH1 and its mutants docked with substrates: (A), D‐LDH1 with PPA; (B), D‐LDH1 with pyruvate; (C), D‐LDH1‐I307L with PPA; (D), D‐LDH1‐I307L with pyruvate; (E), D‐LDH1‐I307M with PPA; (F), D‐LDH1‐I307M with pyruvate. The carbon atoms of NADH are shown in yellow, substrate docking poses are shown in green, active site residues are shown in grey, and the key residue 307 is shown in pink.

Subsequently, to determine the location and action of amino acid residue Ile307 in D‐LDH1 for binding and reduction reactions on PPA, we performed structural modelling and substrate docking (Figure 9). As shown in Figure 9, residue 307 was located at the entrance of the substrate binding site. For the wild‐type D‐LDH1 (A and B), the small side chain of Ile307 could decrease the steric hindrance at the entrance of PPA. Due to the similar structure of Leu307 with Ile307, I307L in Figure 9C,D shows substrate‐docking structures comparable to that of wild‐type D‐LDH1 in Figure 9A,B. Meanwhile, I307M in Figure 9E shows a narrow space between Met307 and PPA, causing steric hindrance, which results in a decrease in substrate affinity for PPA, as shown in Figure 8A. After mutation of I307M, the increment in the side chain length at Met307 might not only prevent PPA molecule entry into the substrate‐binding pocket except for small molecules such as pyruvate but also increased the closeness between pyruvate and the enzyme. Therefore, the enzyme activity for PPA was decreased, while the enzyme activity for pyruvate was increased accordingly.

## DISCUSSION

The genus *Sporolactobacillus* is a gram‐positive, microanaerobic, homofermentative LAB widely distributed in the environment, such as in the rhizosphere soil, tree bark, and Chinese Baijiu (Liu et al., 2019; Tolieng et al., 2017). *Sporolactobacillus* is a unique LAB with endospores that enhance its tolerance to extreme conditions. This lactate tolerance endows *Sporolactobacillus* with an excellent lactate production capacity (Han et al., 2019). Furthermore, recent studies have reported that *Sporolactobacillus* was found in human faecal samples (Forsell et al., 2017), and its abundance in the vaginal microbiota of healthy women was higher than that in women infected with human papillomavirus (HPV) (Wei et al., 2020). Among the different species of *Sporolactobacillus, S. inulinus* was first isolated from chicken feed and has been used as a model strain for lactate production (Zheng et al., 2017). Moreover, *S. inulinus* has been reported as a potential probiotic with acid and bile tolerance, adherence to Caco‐2 cell lines, and antagonistic effects on pathogens (Huang et al., 2007). However, limited information is available on the characterization and industrial application of *S. inulinus*, especially for PLA production. This study demonstrates that *S. inulinus* ATCC 15538 is a superior candidate for the mass production of pure D‐PLA as it produces optically pure D‐PLA (47 mM) with a rapid conversion rate (94 mM·h^−1^) and high conversion yield (88%).

PLA is a by‐product of microbial metabolism and has versatile applications. In addition to the antimicrobial and immune regulatory functions of PLA, it can be used to synthesize poly‐PLA, which is the raw material for biodegradable plastics (Kawaguchi et al., 2017). Additionally, PLA can be used as a cosmetic additive to reduce wrinkles and as a precursor of Danshensu, a Chinese drug for coronary disease. Moreover, the addition of PLA to animal feed increases the immune response and growth performance of pigs and hens (Kim et al., 2014). The aforementioned applications of PLA have stimulated much interest in the development of new synthesis strategies. Whole cell bioconversion using efficient LDH and PPA is regarded as an economic solution to synthesize pure D‐PLA (Rajanikar et al., 2021). Although LDH has been widely studied for lactic acid synthesis from pyruvate, there are few reports of LDH with better substrate affinity for PPA due to its bulky benzene ring structure (Tian et al., 2021). As shown in Table 2, the enzyme efficiency of D‐LDH1 from *S. inulinus* showed significantly higher values of *k*_*cat*_ */Km* (1336.39 mM^−1^ s^−1^) and *k*_*cat*_ */Km*_(PPA)_ */k*_*cat*_ */Km*_(pyruvate)_ (534556) than other LDHs, clearly indicating that D‐LDH1 from *S. inulinus* is one of the best enzymes for producing PLA with the highest substrate specificity for PPA.

In summary, this study investigated the chemical and structural properties of three D‐LDHs from *S. inulinus* and discovered a key residue for catalytic reactions using a site‐mutagenesis approach. Our results indicate that D‐LDH1 is a promising biocatalyst for the biosynthesis of D‐PLA with low *K*_*m*_ and high enzyme efficiency (*k*_*cat*_ */K*_*m*_). We also discovered that residue 307, which is located at the substrate entrance, plays a crucial role in enzymatic activity. This is the first study to investigate three D‐lactate dehydrogenases from *S. inulinus* for PPA reduction and discover superior D‐LDH1 for the production of optically pure D‐PLA.

## AUTHOR CONTRIBUTIONS

YYC, NSH, and TJK contributed to conception and design of the study. YYC performed the study, validation, and statistical analysis, and THP and HS kindly helped in methodology. YYC wrote the original draft of the manuscript and NSH and TJK reviewed and edited the manuscript. All authors read and approved the manuscript.

## CONFLICT OF INTEREST

The authors declare no competing interests.

## ETHICAL APPROVAL

This study does not contain any studies involving human participants or animals performed by any of the authors.

## Supporting information


**Figure S1** HPLC chiral analysis of PLA produced by whole cells of *Sporolactobacillus inulinus*.
**Table S1.** Oligonucleotides used for gene cloning and site‐directed mutagenesis in this study

## Data Availability

All data generated during this study are included in the manuscript. Additional data are available in Supporting Information.
