# Supplementary material for: Biological characterization of D‐lactate dehydrogenase responsible for high‐yield production of D‐phenyllactic acid in Sporolactobacillus inulinus
Source: Microb Biotechnol. 2022 Aug 3;15(11):2717–29. doi: 10.1111/1751-7915.14125 (PMC9618312; doi:10.1111/1751-7915.14125)
Supplement: Supplementary file 1 — Figure S1 HPLC chiral analysis of PLA produced by whole cells of Sporolactobacillus inulinus. Table S1. Oligonucleotides used for gene cloning and site‐directed mutagenesis in this study [file MBT2-15-2717-s001.docx]

Journal -[Microbial Biotechnology](https://www.scimagojr.com/journalsearch.php?q=19700175206&tip=sid&clean=0)

**Biological Characterization of D-Lactate Dehydrogenase Responsible for High-Yield Production of D-Phenyllactic Acid in *Sporolactobacillus inulinus***

Ya-Yun Cheng, Tae Hyeon Park, Hyunbin Seong, Tae-Jip Kim*, Nam Soo Han*

*Brain Korea 21 Center for Bio-Health Industry, Development, Division of Animal, Horticultural, and Food Sciences, Chungbuk National University, Cheongju, Chungbuk, 28644, Republic of Korea*

*Corresponding author: Nam Soo Han

Phone: 82-43-261-2567; Fax: 82-43-271-4412

E-mail: namsoo@cbnu.ac.kr

*Corresponding author: Tae-Jip Kim

Phone: 82-43-261-3354; Fax: 82-43-271-4412

E-mail: tjkim@chungbuk.ac.kr

**Table S1** Oligonucleotides used for gene cloning and site-directed mutagenesis in this study

| **Oligonucleotides** | **Nucleotide sequences (5’ → 3’)** |
| --- | --- |
| **Cloning primers** |  |
| D-LDH1-F | GGAGATATACATATGAAGCTATTCATGTATGGTGTCCAGG |
| D-LDH1-R | TGCGGCCGCAAGCTTTTTTTTTGCTGGTTCAGGTAG |
| D-LDH2-F | GGAGATATACATATGGCTTTTAAAATTATTGCGTATGGTG |
| D-LDH2-R | TGCGGCCGCAAGCTTTTGAGTGACAGCCGGCTTC |
| D-LDH3-F | GGAGATATACATATGAAAATCATTATGTTCAGCGTT |
| D-LDH3-R | TGCGGCCGCAAGCTTGTTTTCTACAGCTACTTTGTTC |
| pET-21a-F | CATATGTATATCTCCTTCTTAAAGTTAAAC |
| pET-21a-R | AAGCTTGCGGCCGCA |
| **Mutagenic primers** |  |
| pELDH1-F | GTTGAAGGAGCATTAAACAG |
| pELDH1-R | GTTGCGTATCGCGGTTTC |
| D-LDH1_I307M-F | ACGCAACATGGTTGAAGGAGCATTAAACAG |
| D-LDH1_I307M-R | CTTCAACCATGTTGCGTATCGCGGTTTC |
| D-LDH1_I307L-F | ACGCAACCTGGTTGAAGGAGCATTAAACAG |
| D-LDH1_I307L-R | CTTCAACCAGGTTGCGTATCGCGGTTTC |

* The underlined regions are nucleotides used for homologous recombination.


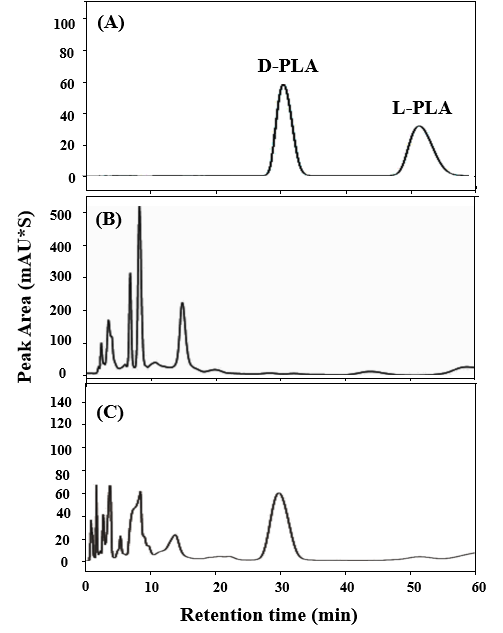


**Figure S1.** HPLC chiral analysis of PLA produced by whole cells of *Sporolactobacillus inulinus*. (A) Standards of D-PLA and L-PLA; (B) Control before cell addition at 0 h; (C) D-PLA synthesis by the whole cell (0.02 g mL^-1^ freeze dry cell mass) of *Sporolactobacillus inulinus* in 50 mM Tris-HCl buffer (pH 8) containing 70 mM PPA and 275 mM glucose at 30℃ for 3 h.
